# LGR5 regulates gastric adenocarcinoma cell proliferation and invasion via activating Wnt signaling pathway

**DOI:** 10.1038/s41389-018-0071-5

**Published:** 2018-08-09

**Authors:** Xiangfei Wang, Xiumin Wang, Yang Liu, Yating Dong, Yanan Wang, Muzaffer Ahmad Kassab, Wufang Fan, Xiaochun Yu, Chen Wu

**Affiliations:** 1 College of Life Sciences, Hebei University, Baoding, 071002 Hebei China; 2 Affiliated hospital of Hebei University, Baoding, 071002 Hebei China; 3 Department of Cancer Genetics and Epigenetics, Beckman Research Institute, City of Hope, 1500 E. Duarte Rd, Duarte, CA 91010 USA

## Abstract

LGR5 plays a critical role in tissue development and the maintenance of adult stem cells in gastrointestinal tract. However, the oncogenic role of LGR5 in the development of gastric adenocarcinoma remains elusive. Here, we show that LGR5 promotes gastric adenocarcinoma cell proliferation and metastasis. We find that knock down of LGR5 or suppression of Wnt signaling pathway by inhibitor C59 arrests gastric adenocarcinoma cell proliferation and invasion. Moreover, treatment of Wnt3a, the activator of Wnt signaling pathway, partially recovers the proliferation defect observed in LGR5 knockdown gastric adenocarcinoma cells. Moreover, LGR5 facilitates β-catenin nuclear accumulation, a surrogate marker of the activation of Wnt signaling pathway. In addition, C59 treatment suppresses transcription of Axin2 and TCF1, both of which are the target genes of β-catenin in gastric adenocarcinoma cells. Gastric adenocarcinoma cells with overexpressed LGR5 form a large quantity of visible actin filaments and pseudopods, suggesting that LGR5 significantly enhances the ability of cell movement, which might capacitate gastric adenocarcinoma cells with enhanced LGR5 expression to gain invasive and migratory properties. Taken together, our results show that LGR5 contributes to cell proliferation and invasion through the activation of Wnt/β-catenin-signaling pathway in gastric adenocarcinoma cells.

## Introduction

Gastric cancer is the fourth most common cancer and the second leading cause of cancer-related deaths^1^. Although few reliable diagnostic biomarkers have been identified for gastric cancer, they cannot be used for the early onset diagnostic purposes. This shortfall contributes to gastric cancer diagnosis at advanced stages with extremely poor prognosis. Moreover, the molecular mechanism of gastric cancer remains elusive, which restricts the use of the personalized treatment in gastric cancer patients.

The leucine-rich G-protein-coupled receptor 5 (LGR5) belongs to the glycoprotein hormone receptor super-family, characterized by presence of a large leucine-rich extracellular domain and the N terminal of the peptide^2^. LGR5 modulates signaling through Wnt pathway upon binding to its cognate ligand R-spondin. Extracellular binding of R-spondins triggers conformational changes in the tyransmembrane domain and consequently activation of downstream signaling cascade including LGR5 itself, buildup in β-catenin which in turn constitutively activates β-catenin dependent transcription^2–4^.

LGR5 expression is elevated in a plethora of cancer types and contributes to cancer phenotype including invasion, migration, and tumorigenicity. For example, in thyroid cancer, overexpression of LGR5 is directly associated with strength, aggressiveness, progression, and metastasis^5^. Moreover, LGR5 expression directly correlates with the tendency of developing colorectal cancer and thus can be substantiated as a potential biomarker^2^. A recent study suggests the presences of a special niche of stem-like cells in colorectal cancer with elevated LGR5 expression suggestive of its potential role in metastasis^6^.

Moreover, LGR5 expression through its downstream Wnt signaling pathway promotes tumor cell proliferation, especially in breast and cervical cancers^7,8^. However, one report by Walker et al. suggests that LGR5 acts as a negative regulator of tumorigenicity, and antagonizes Wnt signaling through its negative regulation of cell adhesion in colorectal cancers^9^. This LGR5-dependent negative regulation specifically restricts colon stem cells to their niche, and loss of LGR5 concomitant with activated Wnt signaling may contribute to the invasive phenotype of colorectal carcinomas^9^. Although, these are conflicting reports regarding the role of LGR5 in progression of tumorigenicity, our previous report along with studies from many other groups have deciphered in detail its role as a marker of stemness in the GI tract. The huge proliferation potential of intestinal tract is largely contributed to the presence of actively proliferating LGR5-positive cryptic base columnar cells^2^. However, the enormous proliferation needs to be regulated in order to prevent the hyperproliferation of the intestinal cells. This is achieved by signaling cascades which directly affect LGR5-positive stem cells^10,11^. Notwithstanding, molecular mechanism of LGR5-mediated tumor metastases remains elusive.

Here, we aim to find the role of LGR5 in tumor cell proliferation and metastasis in gastric cancers. Our results reveal that LGR5 is a positive regulator of cell proliferation, motility, and invasion which are attributed to its indispensible role in regulating cytoskeletal reorganization and Wnt responses in gastric cancer cells.

## Results

### LGR5 expression influences gastric adenocarcinoma cell proliferation

**Fig. 1:**
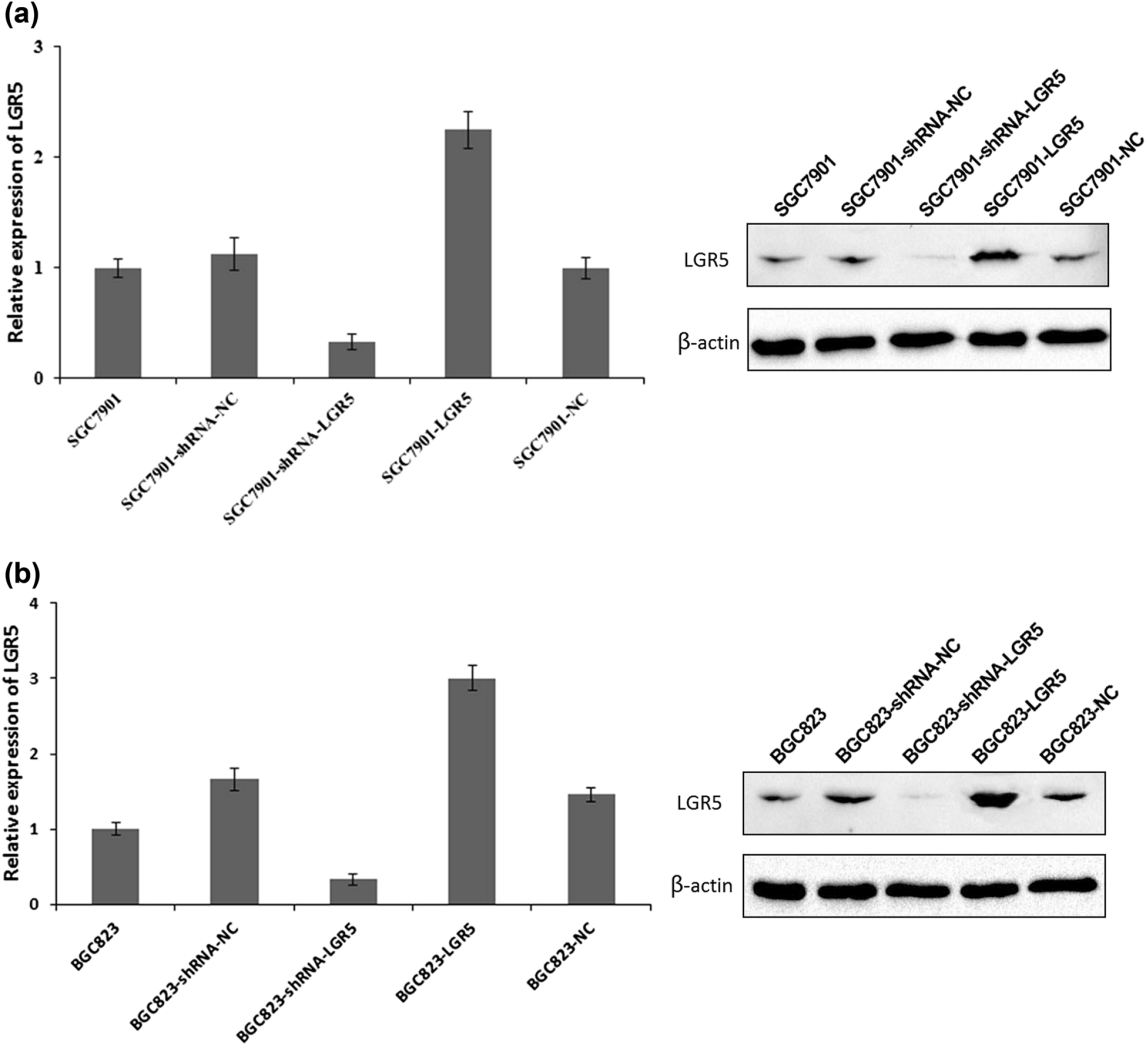
Overexpression and knockdown efficiency of LGR5 were analyzed by western blot. SGC7901 (**a**) or BGC823 (**b**) cells were treated with pGPU6/GFP/Neo containing shRNA to NC sequences, to LGR5 targeting sequence or with pReceiver-M45-LGR5 or pReceiver-M45 as a control. Expression of LGR5 was assessed by western blot (right panels) 72 h after transfection. The band densities were measured by NIH Image J (left panels). The expression levels of LGR5 in parental SGC7901 and BGC823 were considered as “1”

To investigate the biological significance of LGR5 in gastric adenocarcinomas, we used two gastric adenocarcinoma cell lines SGC7901 and BGC823. The cells were transiently transfected with pGPU6/GFP/Neo- shRNA-LGR5, pGPU6/GFP/Neo-shRNA-NC, pReceiver-M45-LGR5, and pReceiver-M45-NC respectively, which were named as SGC7901-shRNA-LGR5, SGC7901-shRNA-NC, SGC7901-LGR5, SGC7901-NC and BGC823-shRNA-LGR5, BGC823-shRNA-NC, BGC823-LGR5, BGC823-NC. The expression of LGR5 in transiently transfected cells was determined by Western blot. The result showed that levels of LGR5 were markedly upregulated in SGC7901-LGR5 and BGC823-LGR5 cells, and downregulated in SGC7901-shRNA-LGR5 and BGC823-shRNA-LGR5 cells (Fig. 1a, b).

**Fig. 2:**
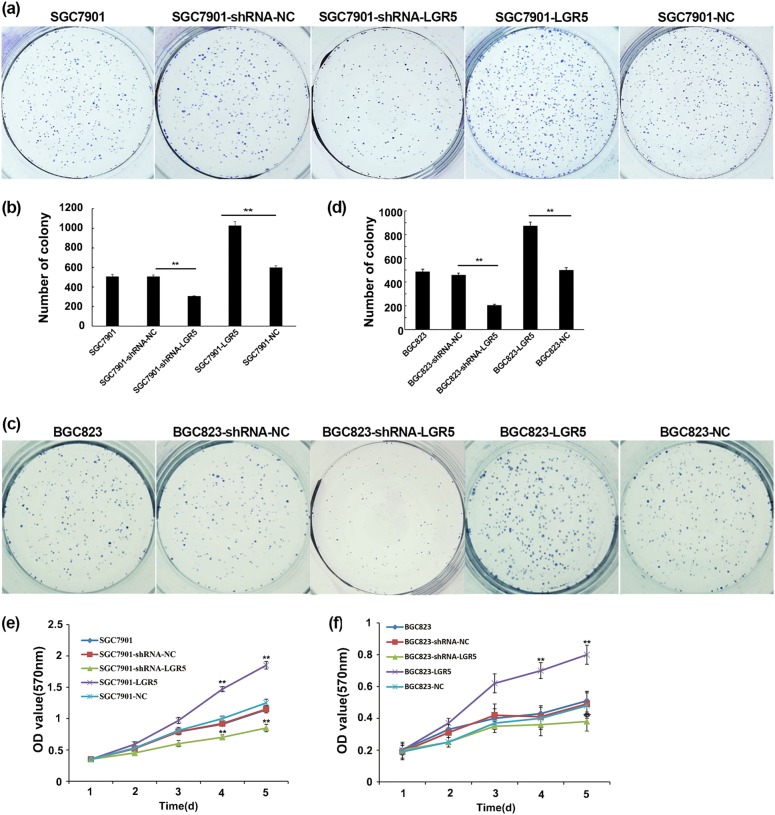
Overexpression and knockdown of LGR5 have opposite effects on cell proliferation. **a**, **c** Gastric adenocarcinoma cells were plated 24 h after transfection with 1.5 × 10^3^ cells/well in six-well plates for 14 days then tested for their ability of clonogenicity as described in Methods. The colonies (≥50 cells) were numbered. Representative images of colonies in plates stained with Giemsa. Images were taken using a Nikon 90i with a DXM 1200C camera. **b**, **d** Data are presented as values of mean ± SD from three independent measurements and the asterisk indicates statistical significance compared with the control (untransfected) parental cells. *P*-values were calculated with Student’s *t* test. **P < 0.01. **e**, **f** Cell viability was assessed by MTT assay. Cell growth curves were determined by absorbance at 570 nm. Experiments were done in triplicate and data from three independent experiments are shown. **P < 0.01: error bars show SEM

We had previously observed that LGR5 was differentially expressed in gastric adenocarcinomas^12^, so we asked if LGR5 regulated gastric cancer cell growth. We performed colony formation assays and MTT assays with LGR5-overexpressed and knockdown in gastric adenocarcinoma cells. It appeared that overexpression of LGR5 had remarkable impact on the gastric adenocarcinoma cell growth. The number of LGR5-overexpression cell colony was significantly more than that of control groups. However, the proliferation ability of shRNA-LGR5 cells was markedly suppressed (Fig. 2a, c). The results on colony formation were consistent in both gastric adenocarcinoma cell lines (Fig. 2b, d). Cell growth was also assessed by MTT assays to further confirm the effect of LGR5 on cell viability. The growth curves of SGC7901-LGR5 or BGC823-LGR5 cells were significantly higher than that of control groups. In contrast, the cells growth of SGC7901-shRNA-LGR5 or BGC823-shRNA-LGR5 started decreasing starting from day 3 (Fig. 2e, f). These results suggest that LGR5 promotes gastric adenocarcinoma cell proliferation.

Moreover, we also stably overexpressed or knockdown LGR5 in SGC7901 and BGC823 (Supplemental Fig. S1), and performed the clonogenic assays. Similar results were observed (Supplemental Fig. S2).

### The role of LGR5 in gastric adenocarcinoma cell invasion

**Fig. 3:**
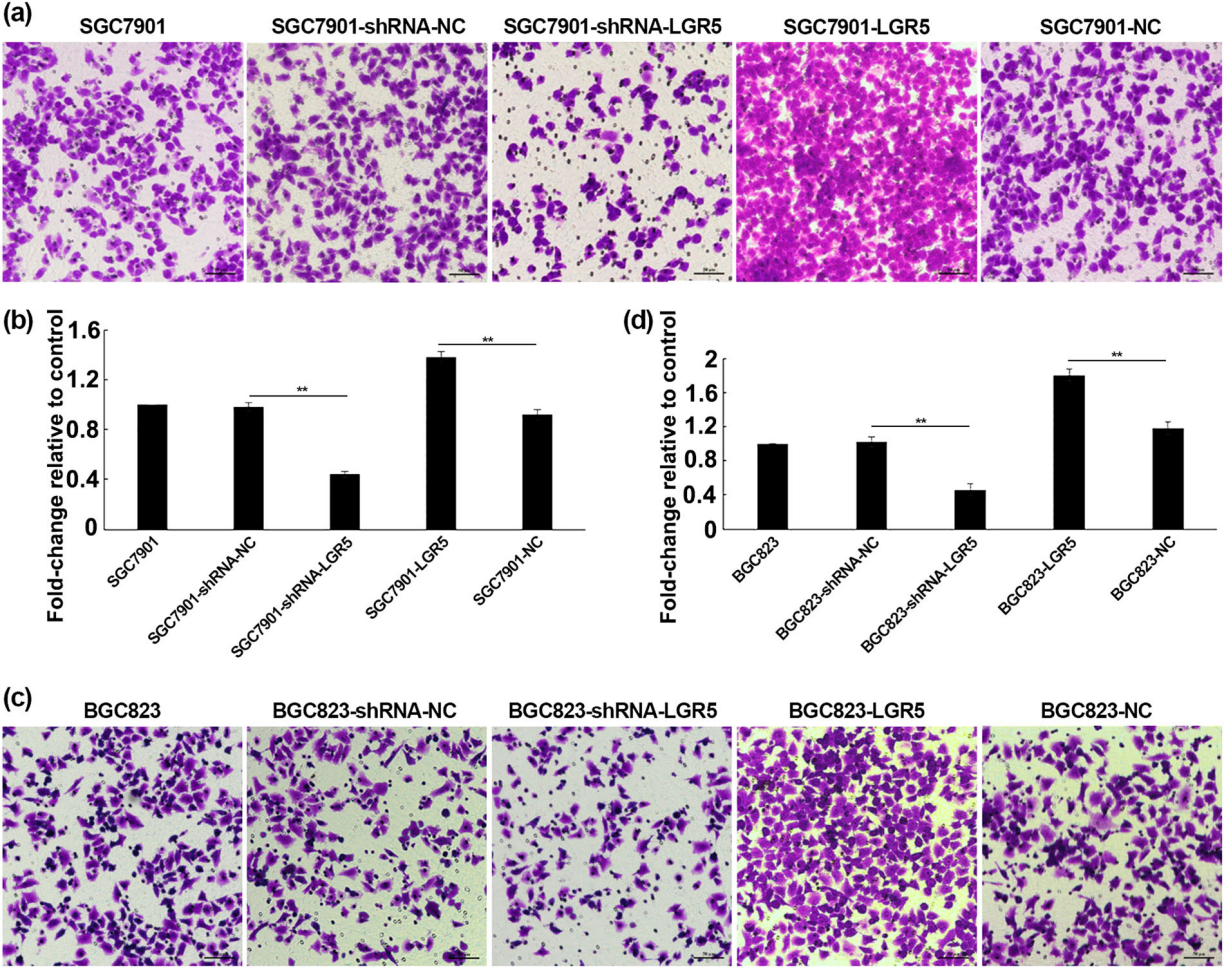
Effects of LGR5 modulation on invasion by transwell assay. **a**, **c** Cells were seeded in Transwell chambers (8 μm pore size) 24 h after transfection, samples were harvested after additional 24 h. Migrated cells were fixed and stained with Crystal Violet. Cells presented on the underside of the filters were counted by a light microscope (Olympus) at ×200 magnification. **b**, **d** Transmigration cells were counted for each of the indicated cells. The graph presented mean ± SD of three separate samples for each cell type; the parental cells were used as the control. Significance levels were determined by the Student’s *t*-test. **P *<* 0.01; *n* = 3

**Fig. 4:**
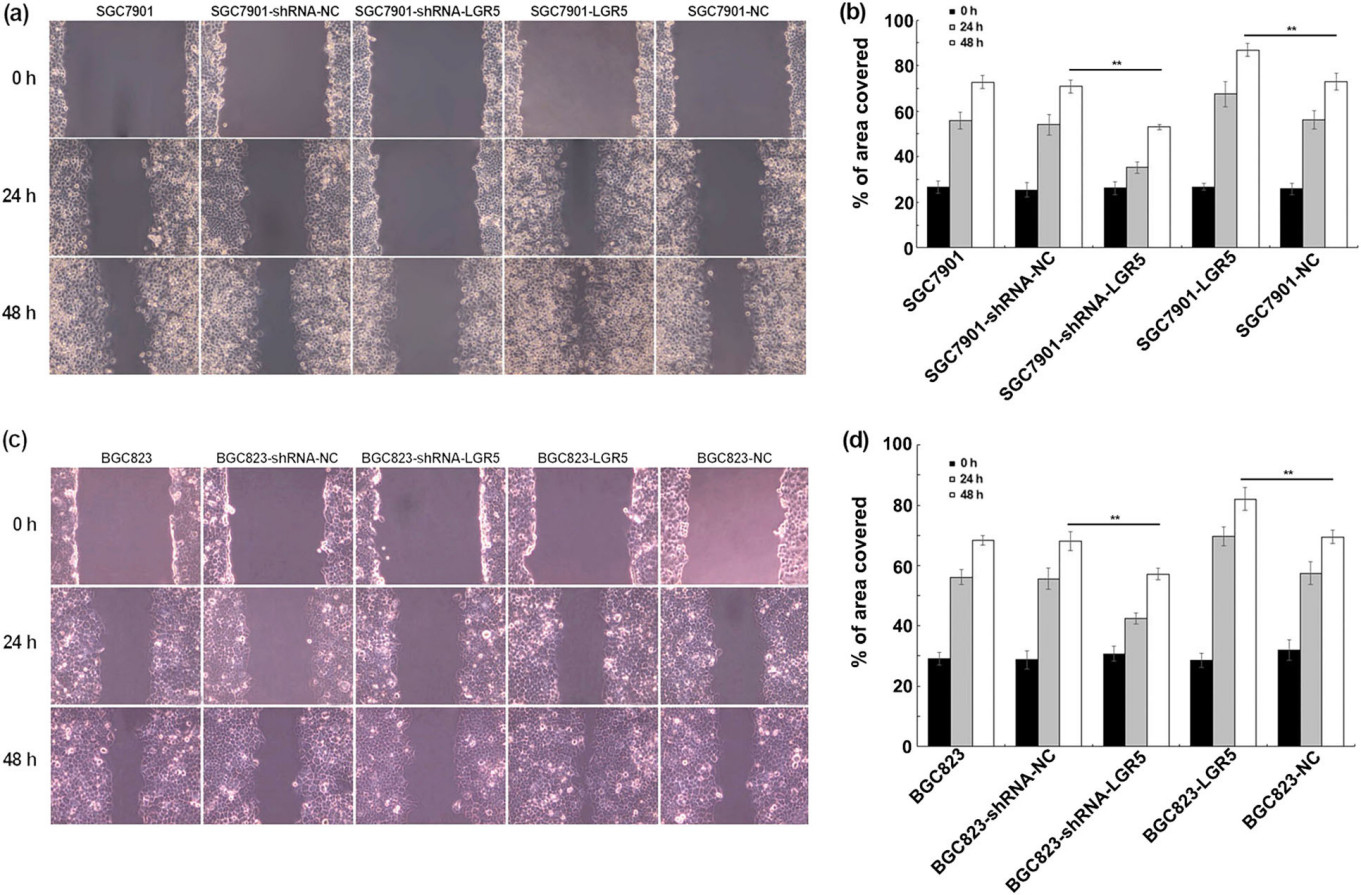
Effect of LGR5 on cell migration in gastric adenocarcinoma cell lines (SGC7901 and BGC823). **a**, **c** SGC7901 and BGC823 cells were transiently transfected with pGPU6/GFP/Neo-shRNA-LGR5, pGPU6/GFP/Neo-shRNA-NC, pReceiver-M45-LGR5, and pReceiver-M45-NC respectively. After 24 h transfection, the cells were grown to 80 % confluence at which time cells were removed with a 10 μl tip in the adherent monolayers. The cells were recovered for 48 h. Images were acquired at 0, 24, and 48 h following the recovery with Olympus digital camera, and analyzed by Image J to measure ability of the cells to repair the wound. **b**, **d** Cellular migration was quantified and represented graphically with results expressed as mean ± SD of three independent measurements. **P *<* 0.01

We next evaluated the role of LGR5 in the invasion and migration of gastric adenocarcinoma cells. As shown in Fig. 3a, c downregulation of LGR5 by shRNA produced markedly inhibited cell invasion in SGC7901 and BGC823 cells as determined by Matrigel in transwell assay, with the relative rate of invasion at 0.44 and 0.46, respectively (**P < 0.01). In contrast, LGR5-overexpressing cells exhibited a higher invasive ability than the control groups, with the relative rate of invasion at 1.38 and 1.81, respectively (Fig. 3b, d). Wound healing assay showed that LGR5 overexpression promoted mobility of gastric adenocarcinoma cells; whereas knockdown of LGR5 resulted in a significant decrease in cellular migration (Fig. 4). Thus, our findings suggest that LGR5 facilitates the motility and invasiveness of gastric adenocarcinoma cells.

### LGR5 regulates gastric adenocarcinoma cell proliferation and invasion via Wnt/β-catenin-signaling pathway

**Fig. 5:**
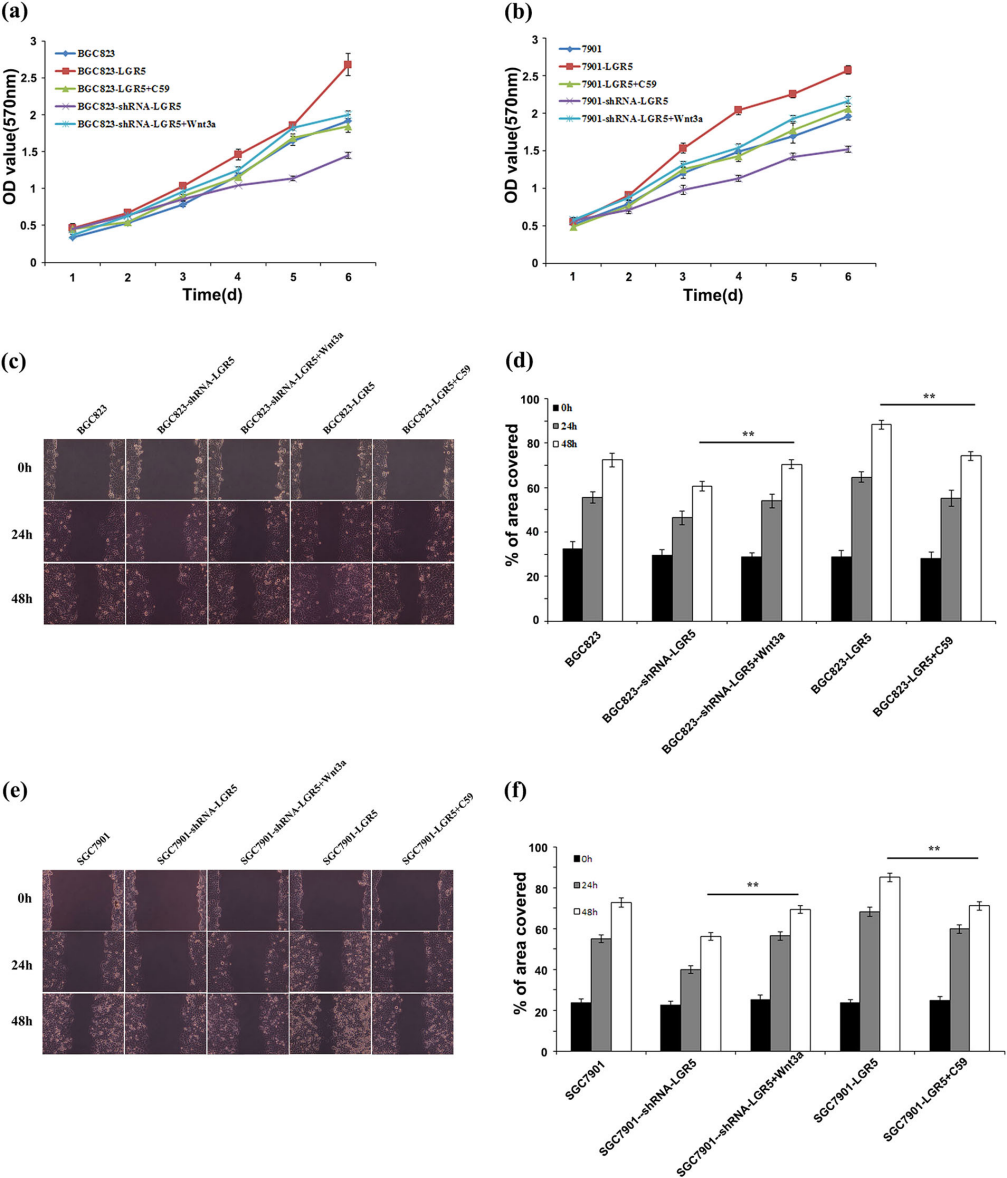
LGR5 regulates proliferation and invasion through Wnt/β-catenin signaling in BGC823 and SGC7901 cells. Cells were transiently transfected with corresponding plasmids. Meanwhile, LGR5 overexpressing cells were treated with Wnt inhibitor C59 and LGR5-knowdown cells were treated with activator Wnt3a. Twenty-four hours after treatment, the cells were examined in the wound healing assay. MTT assays were also started 24 h after treatment. MTT assay (**a**, **b**) and wound healing assay (**c**–**f**) were performed as described in Methods. **P *<* 0.01

It has been reported that LGR5-dependent Wnt/β-catenin-signaling pathway promotes cell proliferation, tumor formation and cancer progression in colorectal cancer, breast cancer and cervical cancer cells^7,8,13^. Here, we examined whether this applied in gastric adenocarcinoma cells. To investigate if LGR5 regulates the gastric adenocarcinoma cell proliferation through Wnt signaling, we used Wnt inhibitor or activator to regulate the Wnt pathway and detected the functional relationship between LGR5 and the Wnt/β-catenin pathway in BGC823 and SGC7901 cells. As shown in Fig. 5a, compared to mock treated BGC823-LGR5 cells, cell proliferation was substantially dampened in BGC823-LGR5 cells treated with Wnt inhibitor C59. In contrast, activation of the Wnt pathway by Wnt3a partially rescued the proliferation defect observed in LGR5 knockdown cells. Similar results were also observed in SGC7901 cells (Fig. 5b).

We further explored the effect of LGR5-dependent Wnt/β-catenin-signaling pathway on gastric adenocarcinoma cell migration. When BGC823-LGR5 or SGC7901 cells were treated with Wnt inhibitor C59, the LGR5-induced migration was suppressed. In contrast, Wnt3a treatment partially rescued the migration defect observed in LGR5 knockdown cells (Fig. 5c–f). These results suggest that LGR5-induced gastric adenocarcinoma cell migration via Wnt signaling pathway.

**Fig. 6:**
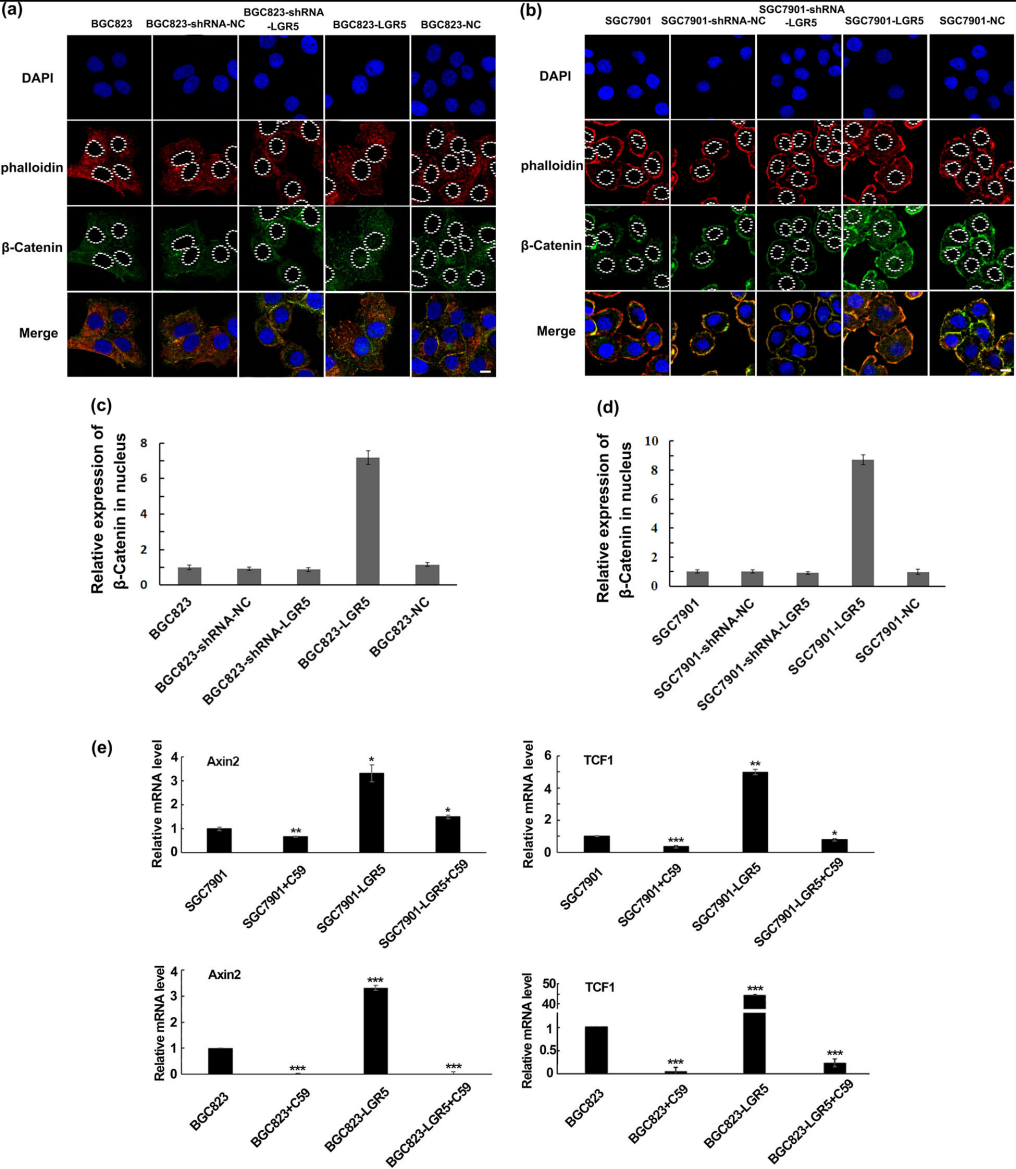
LGR5 regulates expression and location of β-catenin and expression of Wnt/β-catenin target genes in BGC823 and SGC7901 cells. **a**, **b** BGC823 and SGC7901 cells were treated with corresponding vectors. Seventy-two hours after transfection, the cells were subjected to immunofluorescent staining for β-catenin (green) and phalloidin staining (red). Nuclei were visualized with DAPI (blue). In control cells (BGC823, BGC823-shRNA-NC and BCG823-NC; SGC7901, SGC7901-shRNA-NC, and SGC7901-NC), β-catenin was with moderate staining distributed in the cytoplasm. When LGR5 was overexpressed, β-catenin staining was increased in cytoplasm and translocated to the nucleus in BCG823 cells. Depleting LGR5 reduced β-catenin level in nucleus and cytoplasm. Scale bar = 10 μm. **c**, **d** The relative expression level of β-catenin in nucleus in each group was examined by average fluorescence intensity, and the results are presented graphically as mean ± SD of three independent measurements. **P *<* 0.01. **e** Two Wnt/β-catenin target genes were chosen to investigate whether Wnt pathway is activated by LGR5. Axin2 and TCF1 mRNA expression levels in SGC7901 and BGC823 cells treated with or without 10 μM C59 were detected. The graph presented mean ± SD of three separate samples for each cell type, each normalized to parental cells. Significance levels were determined by the unpaired *t*-test. *P < 0.05, **P < 0.01, ***P < 0.001 by *t* test; *n* = 3

Based on the findings that differential regulation of Wnt signaling pathway affected LGR5-induced proliferation and migration in gastric adenocarcinoma cells, we further explored the effect of LGR5 on the localization of β-catenin and the downstream effectors of Wnt signaling pathway in both gastric adenocarcinoma cells. β-catenin is an integral structural component of cadherin-based adherens junctions as well as a key nuclear effector of canonical Wnt signaling in the nucleus. Nuclear β-catenin is a hallmark of Wnt signaling and regulates diverse cellular processes. Activation of the Wnt pathway leads to nuclear accumulation of β-catenin and gene transactivation, an important step in cancer progression. Here, we examined the expression and location of β-catenin in LGR5-overexpressed or knockdown BGC823or SGC7901 cells using immunofluorescence assays. As shown in Fig. 6a, there was an increase of β-catenin expression in the cytoplasm and nuclear fraction in LGR5-overexpressing SGC7901 and BGC823 cells, compared to the control cells. Depletion of LGR5 reduced β-catenin level in nucleus and cytoplasm (Fig. 6a, b). The nuclear level of β-catenin was examined by fluorescence intensity (Fig. 6c). The results indicate that LGR5 induces the accumulation of β-catenin in the nucleus and targets the Wnt/ β-catenin-signaling pathway by regulating the expression level and nuclear translocation of β-catenin. To investigate whether Wnt pathway is activated by LGR5, we further examined two β-catenin targeting genes, Axin2 and TCF1, in SGC7901, SGC7901-LGR5, BGC823, and BGC823-LGR5 cells. As shown in Fig. 6d, the expression levels of both Axin2 and TCF1 were significantly elevated in SGC7901-LGR5 and BGC823-LGR5 cells. Moreover, when these cells were treated with 10 μM C59 for 4 h, C59 significantly inhibited mRNA expression level of Axin2 and TCF1 in LGR5-overexpressing cells compared to control groups (Fig. 6d). Taken together, these results indicate that LGR5 functions through Wnt signaling pathway.

### Effects of LGR5 suppression and overexpression on cell morphology and mobility

**Fig. 7:**
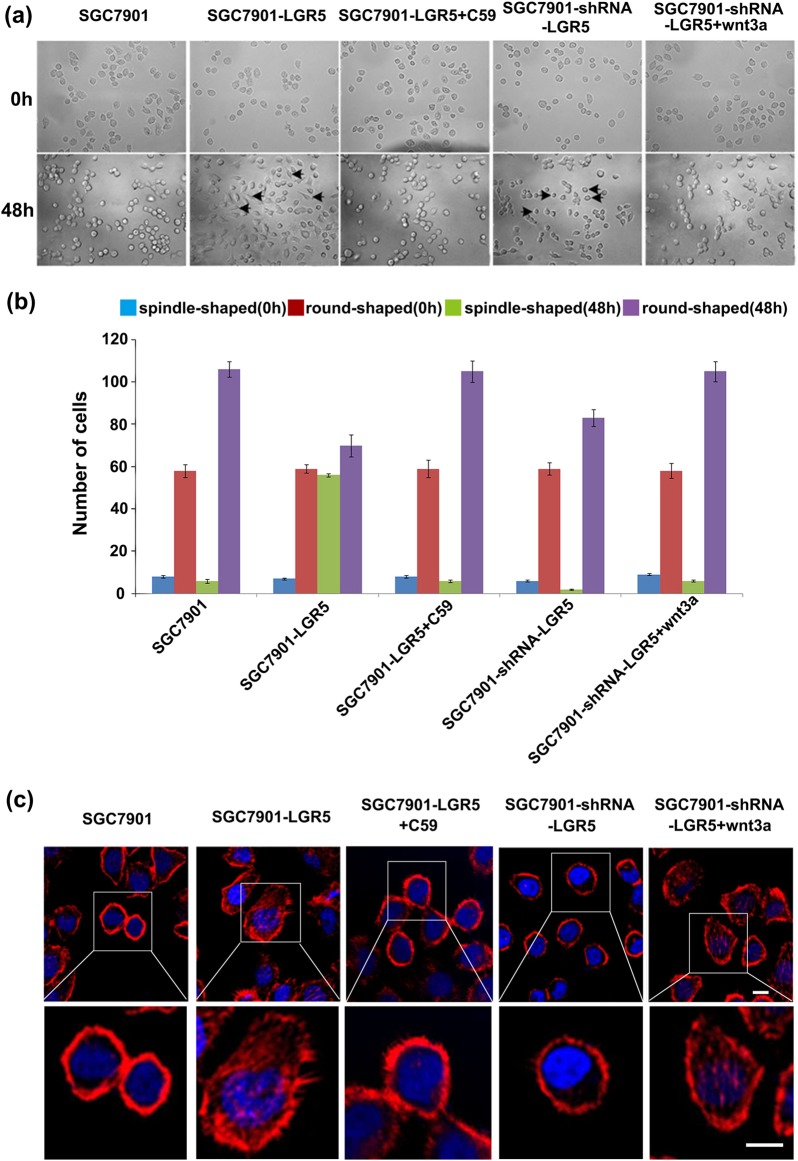

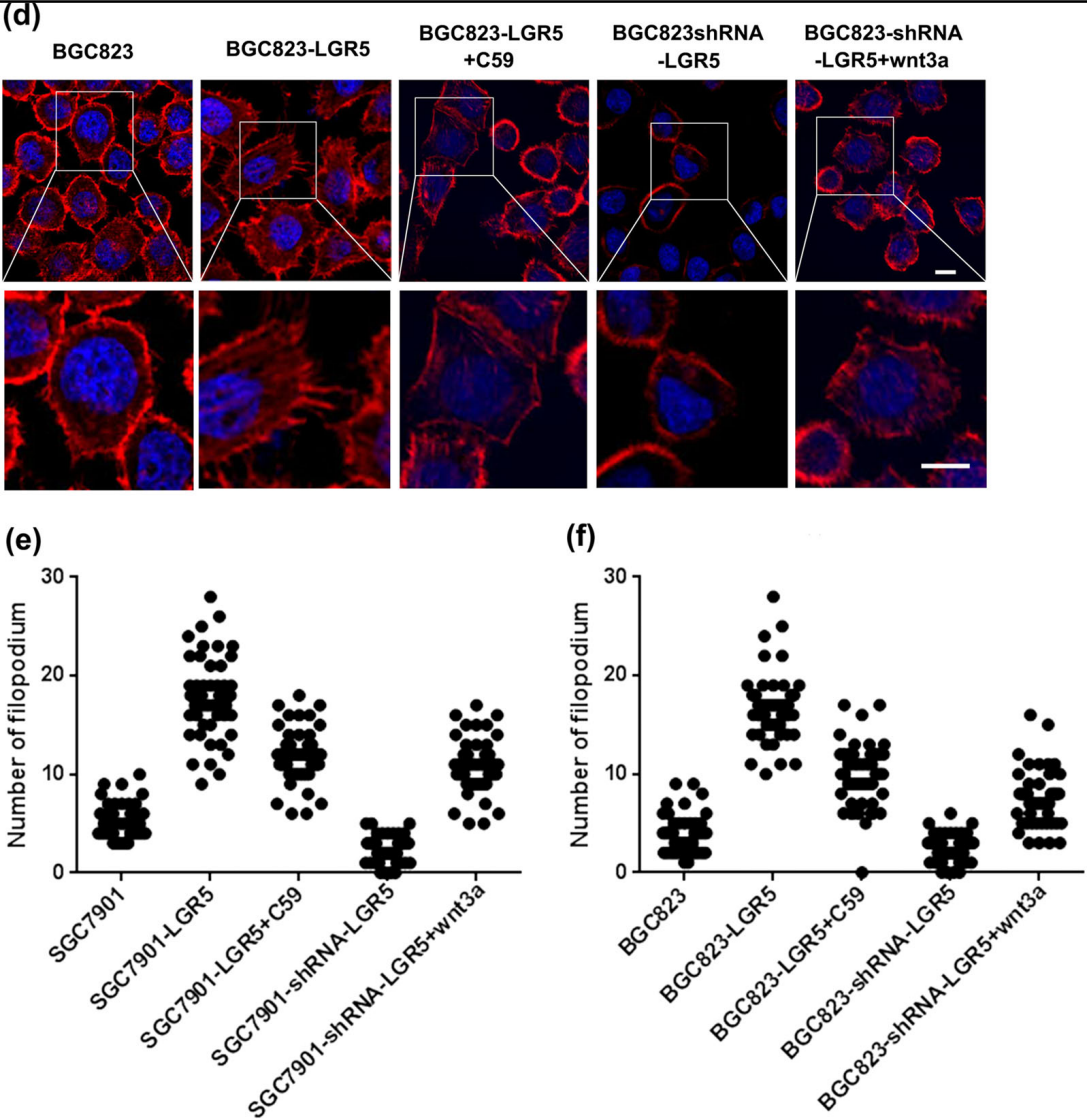
LGR5 overexpressing induced morphological changes. Morphology of SGC7901-LGR5 cells was evaluated by phase-contrast microscopy. Overexpression of LGR5 promotes cell morphological changes, acquiring fibroblast-like appearance with spindle shape (**a**, **b**). Forty-eight hours after transfection, the number of spindle shape cell of SGC7901-LGR5 is significantly more than that of SGC7901-shRNA-LGR5 and control groups. The number of spindle shape and round shape cells in SGC7901-LGR5 is more than that of SGC7901-shRNA-LGR5 and control groups. The SGC7901-LGR5 and SGC7901-shRNA-LGR5 were treated with C59 and Wnt3 to restore the cellular phenotypes. **c**, **d** Rhodamine phalloidin staining of actin filament in gastric adenocarcinoma cells SGC7901 and BGC823. Cells were grown to semi-confluency on poly-l-lysine/laminin, coated, fixed, and stained with rhodamine phalloidin as described in Methods. The actin stress fibers (red) were stained throughout the cells. The cell nuclei were counterstained with DAPI (blue). LGR5 overexpression and knockdown cells were also treated with C59 and Wnt3 to restore the cellular phenotypes. Images of close-ups on pseudopods were in the lower panel. Scale bar = 10 μm

To further examine the molecular mechanism by which LGR5 regulates gastric adenocarcinoma cell invasion, we examined the role of LGR5 in cytoskeleton organization. Cytoskeleton is a highly dynamic structure, the reorganization of which is closely linked with cell morphology and mobility. Generally, cytoskeleton reorganization can trigger microfilament structural changes and increase the number of pseudopodia (lamellipodia and filopodia), which is responsible for cancer cells invasive and migratory properties^14^. Moreover, it has been shown that Wnt signaling, such as Wnt3a mediates these cellular morphological changes^15–18^. Under light microscopy, parental SGC7901 is in relatively round shape. However, when LGR5 was overexpressed in SGC7901, the cells became elongated with fibroblast-like appearance, and we named these cells as spindle shape cells (Fig. 7a). Moreover, the number of spindle shape cells in SGC7901-LGR5 is significantly more than that of SGC7901-shRNA-LGR5 and control groups. The sum of spindle shape and round shape cells in SGC7901-LGR5 is more than that of SGC7901-shRNA-LGR5 and control groups (Fig. 7b). In addition, we treated SGC7901-LGR5 and SGC7901-shRNA-LGR5 with Wnt signaling inhibitor C59 and activator Wnt3 respectively. The treatment restored the cell shape to parental SGC7901, indicating that LGR5 regulates cell morphology via Wnt signaling pathway (Fig. 7a, b).

Since LGR5 changes the cell shape, it is likely to influence cell movement. To test the hypothesis, we assessed the actin remodeling of LGR5-overexpressing or knockdown gastric adenocarcinoma cells using actin staining (phalloidin) assay. We found that LGR5-overexpressing cells formed a large quantity of visible actin filaments and pseudopodia compared with control cells. LGR5-knockdown cells had clear and round cell shape bearing scarcely actin remodeling (Fig. 7c, d). The number of pseudopodia of 50 cells in each group was counted (Fig. 7e, f). The results show that the number of pseudopodia in LGR5-overexpressing cells was markedly higher than that in LGR5-knockdown cells and controls, which suggest that LGR5 might promote cell movement by triggering the microfilament structural change. Moreover, we treated LGR5 overexpression and knockdown cells with C59 and Wnt3a respectively. Both treatments restored the microfilament (Fig. 7c, d), suggesting that LGR5-induced actin remodeling is through Wnt signaling pathway.

## Discussion

LGR5 acts as a stem cell marker and controls tumor progression through its downstream Wnt/β-catenin-signaling pathway. LGR5 overexpression is a signature mark of the stem cells derived from intestinal mucosa, colon, stomach, hair follicle, kidney, and mammary glands^19^. Apart from its crucial role in the maintenance of stemness, the expression profile of LGR5 is directly associated with carcinogenesis and progression in papillary thyroid cancer, colorectal cancer, and breast cancer^5,8,20^. Our previous study indicated that LGR5 expression was correlated with gastric cancer progression^12^. Here, we provided evidence that LGR5 promoted the proliferation, invasion, and migration of gastric adenocarcinoma through activation of Wnt/β-catenin-signaling pathway, which explains the underlying molecular mechanism of LGR5 overexpression during cancer development. In addition, we found that LGR5 increased the number of pseudopodia (lamellipodia and filopodia) of gastric cancer cells, which resulted in an increasing cell motility, thereby promoting cell invasion and migration. This phenomenon was also moderated through LGR5 overexpression-induced Wnt signaling pathway.

In the present study, we demonstrated that LGR5 increased cell proliferation, invasion, and migration using two different gastric adenocarcinoma cell lines SGC7901 and BGC823. In order to regulate Wnt pathway, we used its inhibitor C59 and activator Wnt3a during our studies. C59 is highly potent and cell permeable small-molecule inhibitor of Wnt signaling pathway. C59 exerts its effect by inhibiting the enzymatic activity of PORCN acyltransferase that carries out palmitoylation of Wnts. PORCN-dependent palmotytlation of Wnts is absolutely needed for their activation, secretion, and cognate legend binding^21^. Contrary to C59, Wnt3a activates canonical (β catenin dependent) Wnt signaling pathway that plays a major role in regulating stem cell lineage specification and progenitor cell maintenance^22,23^. We observed that inactivation of the Wnt pathway led to a decrease in LGR5-induced proliferation and migration in gastric adenocarcinoma cells. Wnt3a-mediated rescue could partially recover this proliferation defect observed after LGR5 knockdown. Collectively, this result suggests that LGR5 promotes cell proliferation, invasion and migration through Wnt/β-catenin-signaling pathway in gastric adenocarcinoma cells.

Our observations for gastric adenocarcinoma are in agreement with studies in other cancer types. LGR5 expression is evaluated during cancer cell metastases that can be attributed to both LGR5-positive and -negative carcinomas. LGR5 expression is 6–10-fold higher in metastasis originating from LGR5-positive tumors than that in the negative tumors^6^. Thus, our study elucidates the role of LGR5 in the context of cancer development especially in gastric adenocarcinoma.

We further investigated the relationship between Wnt/β-catenin pathway and LGR5-mediated invasion and migration. β-catenin acts as a crucial downstream molecular target of Wnt/β-catenin-signaling pathway. β-catenin binds with APC and the complex localizes to the leading edge of migratory cells controlling their polarization and migration. This phenomena is attributed to the modulation of cytoskeleton reorganization by the β-catenin–APC complex^24–26^. Upon activation, the level of β-catenin buildup in the cytoplasm, translocates into the nucleus and engages DNA-binding proteins of the Tcf/Lef family. The Tcf/Lef family proteins drives transcriptional of the genes involved in cellular proliferation, invasion and migration^27^. The present study demonstrated that LGR5 not only upregulates the expression of β-catenin, but also affects the subcellular localization of β-catenin in gastric adenocarcinoma cells. Our work indicates that LGR5-mediated Wnt signaling leads to the accumulation and translocation of β-catenin into the nuclei, driving a positive feedback activaton of the Wnt pathway. However, the exact molecular details of this regulation requires further clarification.

Interestingly, our study also indicates that alterations in LGR5 expression for a short period of time is sufficient to reprogram tumor cell growth via Wnt signaling pathway that is in agreement with previous studies^28–30^. Consistently, transient alteration in LGR5 expression led to the nuclear localization of β-catenin and transcriptional induction in its target genes like TCF1 and Axin2. It is likely that upregulation of Wnt target gene expression reprograms tumor cell growth and motility.

To dissect the mechanism of LGR5-mediated gastric tumor invasion, we examined the effect of LGR5 on cell motility by Phalloidin Staining. The formation of pseudopodia is a dynamic process attributed to the local assembly of actin filaments. These pseudopodia are responsible for cancer cell motility^14,31^. We observed that LGR5 induced the formation of vast number of lamellipodia and filopodia in gastric adenocarcinoma cells, corroborating its role in metastasis of gastric adenocarcinoma It was found that LGR5 indeed promoted formation of vast lamellipodia and filopodia as well as actin rearrangement. In conclusion, our results suggest that LGR5 promotes cellular invasion and migration of gastric tumor cells through Wnt/β-catenin-signaling pathway. Further analysis on this pathway in large scale clinical samples may confirm our results. Nevertheless, our results provide the first evidence that overexpression of LGR5 and activation of Wnt signaling pathway are potential biomarker for gastric cancer diagnostics and therapeutic targets for gastric cancer treatment.

## Methods

### Cell culture

Human gastric adenocarcinoma cell lines SGC7901 and BGC823 were acquired from Cell Engineering Research Center of the Fourth Military Medical University. The cells were cultured in Roswell Park Memorial Institute-1640 (RPMI-1640, Life Technologies, Inc., Bethesda, MD) and Dulbecco’s Modified Eagle Medium supplemented with 10% fetal bovine serum (FBS, Gibco), 100 μg/mL penicillin and 100 μg/mL streptomycin. There cells were cultured at 37 °C in a 5 % CO_2_ in a humidified incubator.

### Transfection and shRNA construction

shRNA transfection was carried out using Lipofectamine^®^ 2000 Reagent (Invitrogen) according to the manufactures protocol. Three micrograms of different plasmids were used for transfection of cells grown in a six-well plate at 70% confluency. L LGR5 specific shRNA were synthesized by Shanghai GenePharma Co., Ltd. Shanghai, China. Sequences were as follows: shRNA-LGR5 Forward 5′-CACCGCTCTCATCTTGCTCAATTCCTTCAAGAGAGGAATTGAGCAAGATGAGAGCTTTTTTG-3′; shRNA-LGR5Reverse5′GATCCAAAAAATGTCCATGTCCATATCATATTTCCCTCTCTT GAAGGGAAATATGATATGGACATGGACAC-3′. A scrambled shRNA (shRNA-NC) was also synthesized for the use in control experiments with the following sequence. Forward:5′-CACCGTTCTCCGAACGTGTCACGTCAAGAGATTACGTGACACGTTCGGAGAATTTTTTG-3′ and Reverse:5′-GATCCAAAAAATTCTCCGAACGTGTCACGTAATCTCTTGACGTGACACGTTCGGAGAAC-3′.

The oligonucleotides were annealed and cloned into pGPU6/GFP/Neo-siRNA expression vector (Shanghai GenePharma Co., Ltd., Shanghai, China). The constructed plasmids were confirmed by DNA sequencing. ORF expression clone (EX-Q0041-M45) for LGR5 was purchased from Genecopoeia (Guangzhou, China).

### Real-time quantitative PCR

Total RNA was extracted from the cells using TRIzol reagent (Invitrogen) according to the manufacturer’s protocol. cDNA was synthesized using cDNA Synthesis Kit system (TaKaRa, Japan). The cDNA was used for RT-PCR analysis carried on CFX96^TM^ Real-Time System (Bio-Rad, Hercules, CA, USA). The primers for the RT-PCR were designed using Primer 3 software (Applied Biosystems). The fluorescence of the RT-PCR products was detected with SYBR^®^ Premix *Ex* Taq^TM^ (TaKaRa, Japan). The data were analyzed on CFX Manager software version 3.1 (Bio-Rad) using the 2^−ΔΔCt^ method. The primers used for the RT-PCR were; LGR5, Forward:5′-CTGAACTAAGAACACTGA-3′,Reverse:5′-TTGAGGAAGAGATGAGAT-3′.5′-GAPDH:Forward,GGTCGGAGTCAACGGATTTG-3′,Reverse;5′-ATGAGCCCCAGCCTTCTCCAT-3′.

### Western blot analysis

The cells lysate was prepared in NETN-300 buffer (0.5% Nonidet P-40, 20 mM Tris-HCl, 300 mM NaCl, pH 8.0, 2 mM EDTA. The lysate was boiled in SDS sample buffer and run on SDS-PAGE gel. The proteins were detected using following antibodies: LGR5 mouse monoclonal antibody (Origene, Rockville, MD, USA), β-actin monoclonal antibody (ZSGB-BIO, Beijing, China). The detected proteins were visualized using enhanced chemiluminescence reagent according to standard procedure.

### Colony formation assay

Gastric adenocarcinoma cells were seeded at 1.5 × 10^3^ cells/well in six-well plates and allowed to grow for 14 days. The colonies were washed with PBS, fixed in 4% (w/v) paraformaldehyde and stained with Giemsa (0.4 g/L; Sigma) for colony visualization and counting.

### MTT assay

The effect of LGR5 overexpression and or knockdown on the gastric adenocarcinoma cells was determined by using MTT assay. The cells were grown in 96-well plates at 2 × 10^3^ cells/well and allowed to grow for 24 h after the indicated treatments. A volume of 20 μL MTT Reagent (5 mg/mL) was added to each well and the cells were allowed to grow for 4 h until purple precipitate (formazan) became visible. Then MTT reagent was aspirated, 100 μL DMSO was added to dissolve the formazin precipitate and incubated in dark at room temperature for 15 min. Absorbance values were determined at 570 nm using a multi-channel plate reader.

### Transwell assay

Cell migration assay was performed by using a 24-well transwell chambers with pore size of 8 μM. Cells were transfected with as described above and allowed to grow for 24 h. The upper chamber of the transwell was coated with 40 μL 1:30 mixture of Matrigel (BD Biosciences, Bedford, UK) and dried overnight. Cells were seeded on the upper chamber (1 × 10^5^ cells) in triplicates and allowed to migrate for 24 h at 37 °C in a humidified incubator with 5% CO_2_. After incubation, the non-migratory cells on the upper chamber were removed by a cotton swab. The migratory cells were washed with PBS, fixed in 4% paraformaldehyde and stained with 0.5% Crystal Violet. The migration rate was quantified by analyzing six random fields using a Nikon Eclipse E600 microscope equipped with optical camera CF160 epi-fluorescence and counted.

### Wound repair assays

Cells were cultured and transfected as described previously. Once the cell monolayer reached 80–90% confluence, small scratches were introduced with a sterile 200-μL pipette tip. The cell debris was removed by two washes of PBS and the cells were allowed to grow for the indicated time. The area within the scratch region was examined after every 24 h intervals until the wounds were healed in a group. Images were captured using an inverted phase microscope (Olympus, Japan). The distance migrated by the wound was calculated on photomicrographs, using the same area of the well for each measurement. The migration values were corrected against the proliferation ratio.

### Immunofluorescence

Cells were grown on coverslip and transfected as discussed above and allowed to grow for the time periods indicated. The cells were fixed and permeabilized in 4% paraformaldehyde for 15 min on ice and 0.5% Triton X-100 respectively for 5 min. After blocking with goat serum (Bioss, Beijing, China) for 30 min, they were incubated with anti β-catenin (1:2000, D10A8) antibodies overnight at 4 °C. Next day, the cells were washed with PBS (5 min, 6×) and incubated with with RBITC conjugated secondary antibodies (1:500 dilutions, ZSGB-BIO, Beijing) for 1 h at 37 °C. Non-specific bound antibodies were washed with PBS (5 min, 6×). The nuclei were stained with100 nM DAPI in PBS for 10 min at room temperature. Image capture and analysis of the cells was performed on a laser scanning confocal microscope (LSCM) for immunofluorescence

### Phalloidin staining of cells

The cells were seeded on poly-l-lysine/laminin coated coverslips followed by fixing and permeabilization according to the procedure discussed above. The coverslips were removed at the indicated time points and stained with 100 nM rhodamine phalloidin for 30 min at room temperature. The unbound dye was removed with three PBS washes. The nuclei were stained with DAPI for 10 min at room temperature. The fluorescent images of the actin filaments were then obtained on an inverted phase microscope (Olympus, Japan).

### Time line of the assays

The transfection was done on day 0. Twenty-four hours later, clonogenic formation assays, MTT assays, wound healing assays, and transwells assays were performed. For transwell assays, the imaging and counting were done at day 2 (Fig. 2). The morphological changes of gastric cancer cells were also performed on day 2 (Fig. 7). Wound healing assay was performed at days 1–3 (Figs. 4 and 5), MTT assays at day 5 (Figs. 2e, f and 5a, b) and coloney formation assay at day 14 (Fig. 2a–d). The expression profile of LGR5, Wnt target gene expression (Fig. 6e) and translocation of β-Catenin (Fig. 6a–d) was determined on day 3 (Fig. 1). The inhibitor C59 and the activator were introduced into the culture 12 h post transfection

### Statistical analysis

Each experiment was repeated at least three times. The statistical analyses were performed using SPSS 19.0 (SPSS, Inc., Chicago, IL, USA, 2012). Data were expressed as mean ± SD. The statistical correlation of data between groups was analyzed by Student’s *t* test. *P < 0.05 was considered to be statistically significant.

## Supplementary information

Supplemental Figure legend

Supplemental Figure S1

Supplemental Figure S2
