# Correction: LGR5 regulates gastric adenocarcinoma cell proliferation and invasion via activating Wnt signaling pathway

**DOI:** 10.1038/s41389-019-0118-2

**Published:** 2019-02-02

**Authors:** Xiangfei Wang, Xiumin Wang, Yang Liu, Yating Dong, Yanan Wang, Muzaffer Ahmad Kassab, Wufang Fan, Xiaochun Yu, Chen Wu

**Affiliations:** 1 College of Life Sciences, Hebei University, 071002 Baoding, Hebei China; 2 Affiliated hospital of Hebei University, 071002 Baoding, Hebei China; 3 Department of Cancer Genetics and Epigenetics, Beckman Research Institute, City of Hope, 1500 E. Duarte Rd, Duarte, CA 91010 USA

**Correction to:** ***Oncogenesis*** **7:57;**

10.1038/s41389-018-0071-5; Published online 09 August 2018

The original version of this Article contained an error in the author affiliations.

The second corresponding author Dr. Xiaochun Yu is only affiliated with [3] Department of Cancer Genetics and Epigenetics, Beckman Research Institute, City of Hope, 1500 E. Duarte Rd, Duarte, CA 91010, USA. He is not affiliated with [1] College of Life Sciences, Hebei University, Baoding 071002 Hebei, China.

This has now been corrected in both the PDF and HTML versions] of the Article.

