# Supplementary material for: LGR5 regulates gastric adenocarcinoma cell proliferation and invasion via activating Wnt signaling pathway
Source: Oncogenesis. 2018 Aug 9;7(8):57. doi: 10.1038/s41389-018-0071-5 (PMC6082861; doi:10.1038/s41389-018-0071-5)
Supplement: Supplementary file 1 — Supplemental Figure legend [file 41389_2018_71_MOESM1_ESM.docx]

**Supplemental Information**

**Supplemental Figure Legend**

**Figure S1. Western blot analysis of LGR5 stably overexpressing and knockdown cell lines.** SGC7901 (a) or BGC823 (b) cells were transfected with pGPU6/GFP/Neo containing shRNA to NC sequences, to LGR5 targeting sequence or with pReceiver-M45-LGR5 or pReceiver-M45 as a control. After 24 h, the transfected cells were selected with 800 mg/ mL of G418 (Invitrogen) for 14 days and the media containing G418 were changed every day. Thereafter, the individual cell colonies were transferred to 24-well plates and maintained in the presence of G418 and then pooled for further studies. The cells were harvested and the efficiency of LGR5 overexpression and knockdown was evaluated by Western blot. Expression of LGR5 was assessed by Western blot (right panels). The band densities were measured by NIH Image (left panels).

**Figure S2. Effects of LGR5 on gastric cancer proliferation.** (a and c). LGR5 stably overexpressing and knockdown cells were plated after transfection at 1.5 × 10^3^ cells/well in 6-well plates for 2 weeks then tested for their ability of clonogenicity as described in Materials and Methods. The colonies (≥50 cells) were numbered. Representative images of colonies in plates stained with Giemsa. Images were taken using a Nikon 90i with a DXM 1200C camera. (b and d) Data are presented as values of mean ± SD from three independent measurements and the asterisk indicates statistical significance compared with the control (untransfected) parental cells. P-values were calculated with Student's t test. **P<0.01.
