# Supplementary figures and images for: LGR5 regulates gastric adenocarcinoma cell proliferation and invasion via activating Wnt signaling pathway
Source: Oncogenesis. 2018 Aug 9;7(8):57. doi: 10.1038/s41389-018-0071-5 (PMC6082861; doi:10.1038/s41389-018-0071-5)

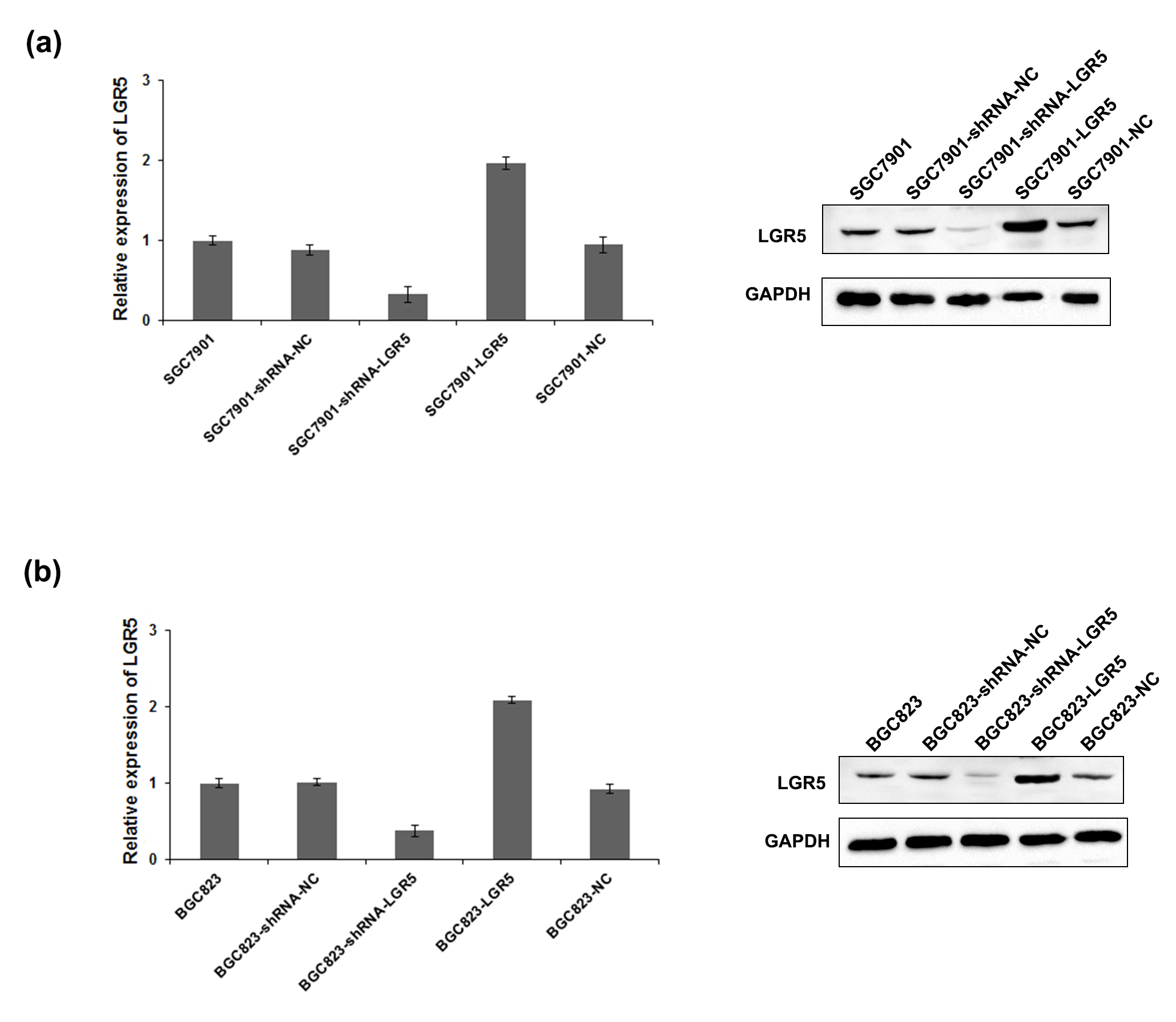

Supplement: Supplementary file 2 — Supplemental Figure S1 [file 41389_2018_71_MOESM2_ESM.tif]

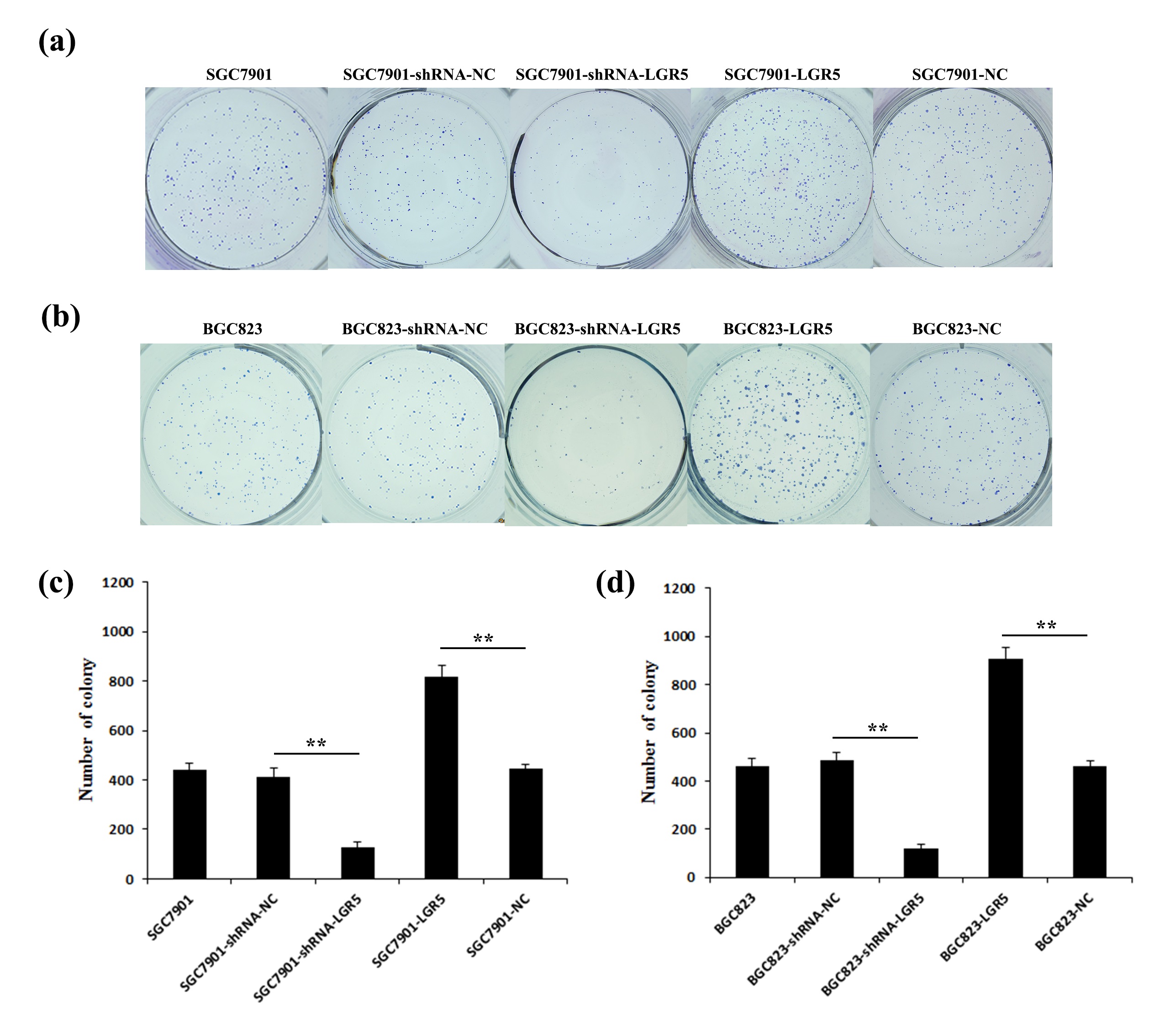

Supplement: Supplementary file 3 — Supplemental Figure S2 [file 41389_2018_71_MOESM3_ESM.jpg]
